# Causal association between inflammatory bowel disease and herpes virus infections: a two-sample bidirectional Mendelian randomization study

**DOI:** 10.3389/fimmu.2023.1203707

**Published:** 2023-07-03

**Authors:** Menglong Zou, Wei Zhang, Lele Shen, Yin Xu, Ying Zhu

**Affiliations:** ^1^ Department of Gastroenterology, The First Hospital of Hunan University of Chinese Medicine, Changsha, Hunan, China; ^2^ Department of Dermatology, The First Hospital of Hunan University of Chinese Medicine, Changsha, Hunan, China

**Keywords:** Mendelian randomization, inflammatory bowel disease, ulcerative colitis, Crohn’s disease, chickenpox, herpes zoster, mononucleosis, causal association

## Abstract

**Background:**

Previous observational or retrospective studies have suggested an association between inflammatory bowel disease (IBD) and herpes virus infections. Using Mendelian randomization (MR) approach, our objective was to determine whether there was a causal association between IBD and herpes virus infections.

**Methods:**

In genome-wide association study (GWAS) datasets of the International Inflammatory Bowel Disease Genetics Consortium, we obtained genetic instrumental variables for three phenotypes from 34,652 participants (12,882 IBD cases and 21,770 controls), 27,432 participants [6,968 ulcerative colitis (UC) cases and 20,464 controls], and 20,883 participants [5,956 Crohn’s disease (CD) cases and 14,927 controls], respectively. Summary statistics for herpes virus infections (chickenpox, herpes zoster, and mononucleosis) were obtained from the FinnGen database. MR results were expressed as odds ratio (OR) with 95% confidence interval (CI).

**Results:**

Our study found no evidence of a causal effect of genetically predicted IBD on herpes virus infections [*P* value for inverse variance weighting (IVW): 0.063 to 0.652]. For the subtypes of IBD, UC had a suggestive association with mononucleosis (*P* value for IVW: 0.023). It appeared that CD was also weakly associated with mononucleosis (*P* value for IVW: 0.058; *P* value for Weighted median: 0.036). In addition, we found a suggestive causality for CD on chickenpox (*P* value for IVW: 0.038). Neither UC (*P* value for IVW: 0.574) nor CD (*P* value for IVW: 0.168) has a causal effect on herpes zoster. The results of the bidirectional MR analysis did not indicate that herpes virus infections were associated with IBD, UC or CD (*P* value for IVW: 0.239 to 0.888).

**Conclusion:**

This study showed a suggestive causality for both CD-chickenpox and UC-mononucleosis, despite no associations reaching a statistical significance value after corrections for multiple testing. There was no evidence of a causal association between IBD and its two subtypes on herpes zoster.

## 1 Introduction

Inflammatory bowel disease (IBD), including two subtypes ulcerative colitis (UC) and Crohn’s disease (CD), is a group of diseases characterized by chronic inflammation of the gastrointestinal tract (1). The clinical symptoms include hematochezia, diarrhea, abdominal pain and weight loss (1). The incidence of IBD is increasing worldwide, particularly in newly industrialized countries such as Africa and South America (2). Many countries in North America, Europe, and Oceania already exceed 0.3% of IBD prevalence, which causes a serious public health burden (2). The pathogenesis of IBD is not fully understood, but factors such as genetics, immunity, environment, and gut microbes are thought to be involved (3). These pathogenetic mechanisms have similarities with other diseases. Briefly, there are shared mechanisms between multiple diseases. For example, there are clear genetic and immunological links between autoimmune rheumatic diseases and inflammatory bowel disease (4). Although a number of observational studies and retrospective studies have analyzed associations between multiple diseases (5–7), high-quality evidence of genetic cause-and-effect associations is still needed.

Herpes virus is a common DNA virus that can cause a number of different infections, including oral and labial herpes, herpes zoster, and chickenpox (8). Herpes virus is usually transmitted by contact with the source of the infection, such as the patient’s skin and saliva. After infection, the virus can latent in the nervous system and reactivate when the immune system is challenged. An abnormal immune response appears to therefore increase the risk of herpes virus infections. In recent years, some evidence has suggested a link between herpes virus infections and IBD (9, 10). A descriptive study by Côté-Daigneault et al. based on health registry data from 1996 to 2015 found a high prevalence of herpes zoster in patients with IBD, particularly in women (11). A meta-analysis showed that patients with IBD had a 1.68-fold increased risk of infection with herpes zoster compared to those without IBD (12). Another study showed up to 79.4% Epstein-Barr virus (EBV) positivity in patients with IBD (13). However, these studies were limited by unmeasured confounding or other biases (e.g., reverse causation). It is unclear whether the association between IBD and herpes virus infections is a casual effect.

Mendelian randomization (MR) is a novel method of epidemiological research that uses genetic variants as instrumental variables to assess the presence or absence of causal effects between exposure and outcome (14). Evidence of causal effects analyzed in this method greatly reduces the bias caused by confounders in observational studies, because the genetic variants are randomly assigned at the time of conception and are unaffected by environmental factors and self-selected lifestyle (15). Large-scale genome-wide association studies (GWAS) data of herpes virus infections and IBD have been published publicly, providing an opportunity to sort out the complex causal relationships between them through MR analysis.

The current studies mainly report that IBD is associated with three herpes virus infections (chickenpox, herpes zoster, and mononucleosis) (11, 13, 16), mainly caused by varicella-zoster virus (VZV) and EBV, respectively. Primary infection with VZV and EBV usually occurs at a young age. However, both viruses remain in the body as latent forms after the primary infection has subsided. Chickenpox is caused by primary VZV infection, whereas herpes zoster is the result of latent VZV reactivation. Primary EBV infection in children usually has no obvious symptoms, whereas mononucleosis is usually the result of infection later in life. In this study, we used a two-sample MR analysis approach to evaluate the causal effect associations between IBD and three active herpes virus infections (chickenpox, herpes zoster, and mononucleosis).

## 2 Materials and methods

**Figure 1:**
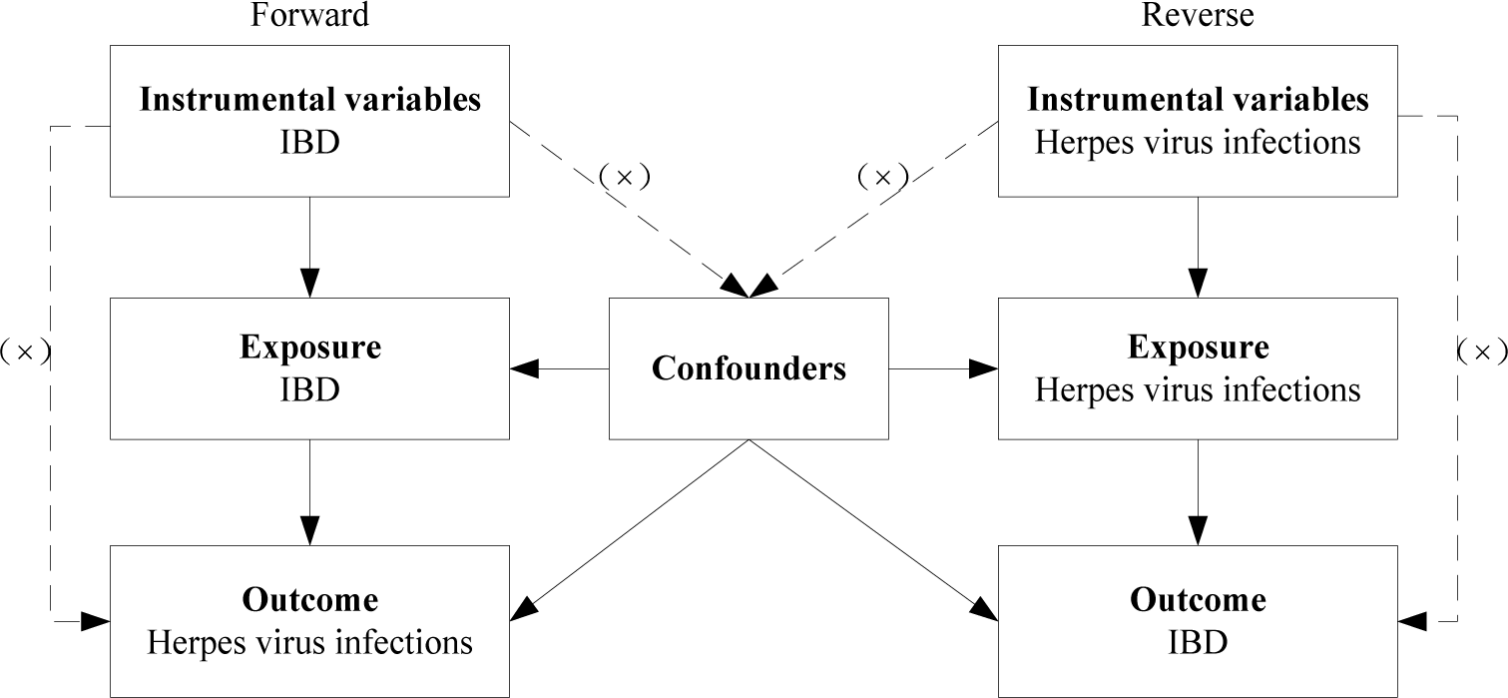
Diagram for three main assumptions of MR study. Lines with arrows indicate that the instrumental variables are associated with the exposure and could only affect the outcome through the exposure. Dashed lines indicate that the instrumental variables are independent of any confounding variables. IBD, inflammatory bowel disease.

Representative phenotypic single-nucleotide polymorphisms (SNPs) were selected as genetic instrumental variables to perform two-sample MR study. The genetic instrumental variables for phenotypes should meet the three main assumptions (**Figure 1**): 1. instrumental variables should be strongly associated with corresponding phenotype; 2. instrumental variables should be unaffected by potential confounders of the exposure-outcome association; 3. direct links between the instrumental variables and the outcome are not available (17). MR analysis was conducted to evaluate bidirectional causality between IBD (including UC and CD subtypes) and herpes virus infections (including chickenpox, herpes zoster, and mononucleosis). Ethical approval was not required as we used publicly available GWAS results from the IIBDGC database, FinnGen database, and UK Biobank in this study.

### 2.1 Data source

Genome-wide association studies (GWAS) summary statistics for IBD was selected from the International Inflammatory Bowel Disease Genetics Consortium (IIBDGC), including 34,652 European-descent participants (12,882 IBD cases and 21,770 controls) (18). In that study, the UC subtype included 27,450 European-descent participants (6,986 diagnosed with UC and 20464 controls), and the CD subtype included 20,883 European-descent participants (5,956 diagnosed with CD and 14,927 controls). In order to avoid sample size overlap, we collected summary statistics for three herpes viruses from the FinnGen database (19). The total number of participants included in the analysis for chickenpox was 331,233, comprising of 1,146 cases and 330,087 controls. For herpes zoster, the total number of participants was 334,307, with 4,220 cases and 330,087 controls. Similarly, for mononucleosis, the total number of participants was 335,814, with 2,099 cases and 333,715 controls.

### 2.2 SNPs selection

The genome-wide statistical significance threshold (*P* < 5×10^-8^) was met for all SNPs to fulfil assumption 1. To obtain independent SNPs, we pruned SNPs in linkage disequilibrium (LD) (r^2^ < 0.001 and distance = 10,000 kb) with the clump data function in the TwoSampleMR package in the R software. LD between all SNPs was based on the European 1000 Genome Project reference panel (20). For the data extraction of IBD, UC, and CD, 65, 39, and 53 relevant SNPs were obtained, respectively. Fewer SNPs were extracted as instrumental variables for herpes virus infections at a threshold of *P* < 5×10^-8^. With a looser significance threshold of *P* < 5×10^-6^ (21), 5 SNPs for chickenpox, 11 SNPs for herpes zoster, and 13 SNPs for mononucleosis were extracted as instrumental variables. Given the low significance threshold, we calculated the *F* statistics to assess the bias of the weak instrumental variables (22). When the *F* statistic > 10, the bias of weak instrumental variables can be ignored (23). The selected SNPs were matched with data from the Phenoscanner database to confirm the fulfillment of assumption 2. A threshold of *P* < 5×10^-6^ was applied to eliminate SNPs that were closely associated with potential confounders (24). Data on exposure and outcome were merged and harmonized by effect alleles. For the merged data, we discarded SNPs that were potentially correlated with the outcome (*P* < 5×10^-6^) due to possible violation of assumption 3. Detailed information regarding instrumental variables for our study can be found in **Supplementary Tables 1–6**.

### 2.3 Immunosuppressants analysis

The use of immunosuppressants as a treatment for patients with IBD (including both subtypes) was identified as a confounding factor in our study, as it may elevate the susceptibility to herpes virus infections. Therefore, we employed MR analysis to evaluate the potential causal relationship between the utilization of immunosuppressants and the occurrence of three herpes virus infections. Subsequently, the instrumental variables of the exposure data were purged of SNPs that exhibited a strong correlation with immunosuppressants (*P* < 5×10^-8^), provided that causal relationships were established previously. The GWAS summary statistics for immunosuppressants based on the UK Biobank, including 164,520 European-descent participants, were obtained from the study by Wu et al. (25). This study was also applied in a recent MR study to assess the impact of immunosuppressants use on the increased risk of Parkinson’s disease in patients with rheumatoid arthritis (26). In a comparable manner, the parameters *P* < 5×10^-8^, r^2^ < 0.001 and distance = 10,000 kb were established to conduct a screening of the instrumental variables for immunosuppressants.

### 2.4 Statistical analysis

According to the MR analysis guidelines (27), the inverse variance weighting (IVW) method is statistically most effective when the instrumental variables meet the three main assumptions. Therefore, the IVW method was used as the primary analysis in the present MR study to assess the causal effect. Two additional MR methods (MR-Egger regression and Weighted median) served as a complement to minimize the presence of confounding by heterogeneity and horizontal pleiotropy (28). MR results were expressed as odds ratio (OR) with 95% confidence interval (CI).

In addition, we performed multiple sensitivity analysis to check the robustness of the MR results, such as Cochran’s Q test, MR-Egger intercept test, MR-PRESSO, leave-one-out analysis, and MR-Steiger test of directionality. Cochran’s Q is a test for heterogeneity and a significant *P* value indicates the presence of heterogeneity (29, 30). MR-Egger intercept analysis was performed to assess horizontal pleiotropy (31). In cases where there was heterogeneity or pleiotropy in the MR results, we used MR-PRESSO to identify potential outliers (28). The MR analysis was re-conducted after removing the outliers. Leave-one-out analysis was conducted to identify if any single SNPs had a disproportionate effect on the estimates (28). The MR-Steiger directionality test was performed to evaluate the validity of the direction of causality (32).

Given the involvement of three autoimmune diseases and three herpes viruses in the analysis, a Bonferroni corrected *P*-value of 0.0056 (0.05/9) was established as the threshold for statistical significance. Suggestive association results were those that were significant (*P* < 0.05) before but not after multiple-comparison correction (*P* < 0.0056). All analysis in our study were performed using the package “TwoSampleMR (version 0.5.6)” and the package “MRPERSSO (version 1.0)” in the Rstudio (R version 4.2.2).

## 3 Results

### 3.1 Causal effects of IBD, UC, or CD on herpes virus infections

Bias due to weak instrumental variables could be ignored as the *F* statistics for all SNPs in IBD and its two subtypes were greater than 10. For immunosuppressants, we identified five SNPs as instrumental variables (**Supplementary Table 7**). Interestingly, we found that genetically predicted use of immunosuppressants did not increase the risk of herpes virus infections (*P* value for IVW: 0.410 to 0.754, **Supplementary Table 8**). Therefore, the bias caused by immunosuppressants could also be negligible in the forward MR analysis.

**Figure 2:**
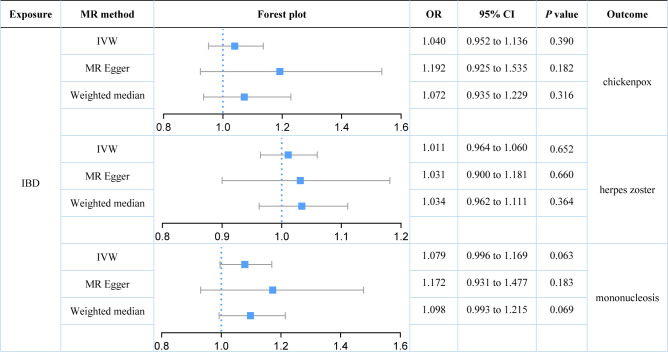
Estimates of the causal relationship between inflammatory bowel disease and each herpes virus infection expressed as an odds ratio (OR) and 95% confidence interval (CI). MR, mendelian randomization; IBD, inflammatory bowel disease; IVW, inverse variance weighting.

Our study found no strong evidence of a causal effect of genetically predicted IBD on herpes virus infections (*P* value for IVW: 0.063 to 0.652, **Figure 2**). The MR-PRESSO method did not detect any outliers that affected the results. The results were consistent with the estimations made in MR-Egger and Weighted median. No evidence of horizontal pleiotropy in the MR-Egger intercept test (*P* value: 0.267 to 0.762). Although Cochran’s Q test indicated that there was some heterogeneity (*P* value: 0.015 to 0.786), the Steiger directionality test suggested that the causal directions were true.

**Figure 3:**
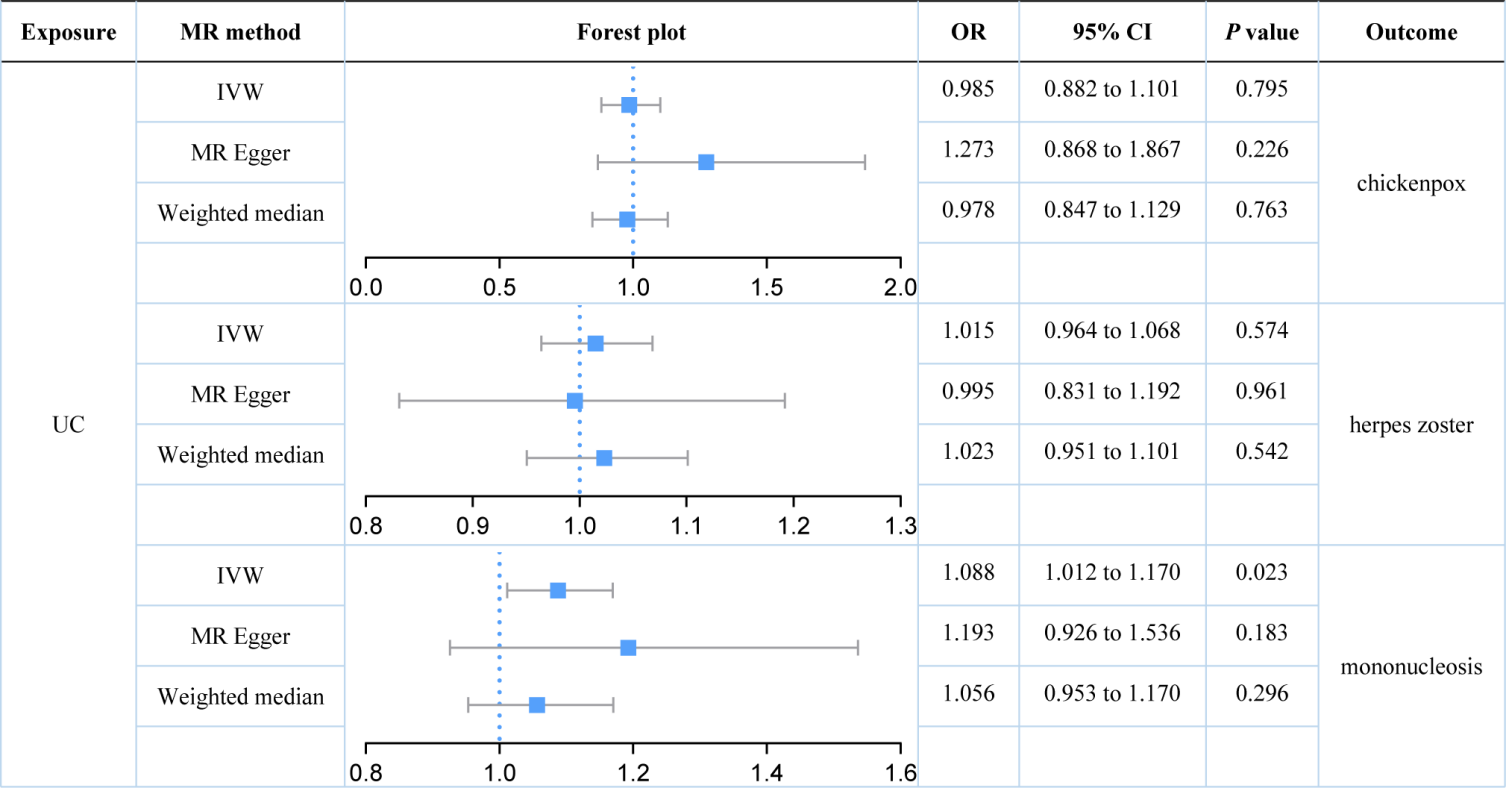
Estimates of the causal relationship between ulcerative colitis and each herpes virus infection expressed as an odds ratio (OR) and 95% confidence interval (CI). MR, mendelian randomization; UC, ulcerative colitis; IVW, inverse variance weighting.

**Figure 4:**
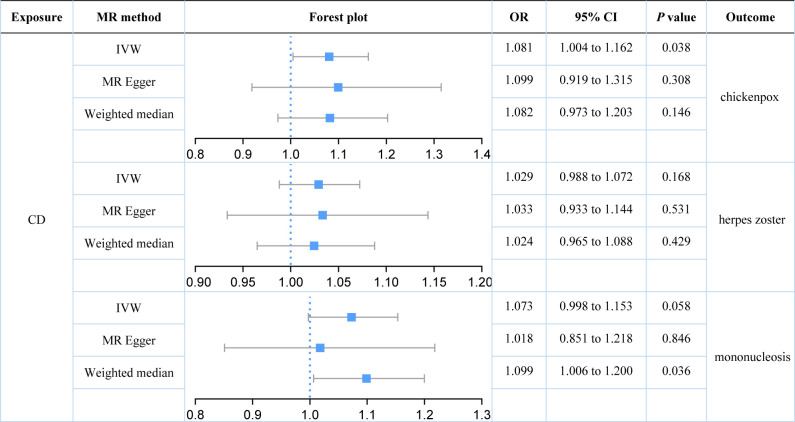
Estimates of the causal relationship between Crohn’s disease and each herpes virus infection expressed as an odds ratio (OR) and 95% confidence interval (CI). MR, mendelian randomization; CD, Crohn’s disease; IVW, inverse variance weighting.

We further explored the causal relationships between two subtypes of IBD (UC and CD) on three herpes virus infections in order to preclude the effects of shared genetic mechanisms. We found an association between UC and mononucleosis (*P* value for IVW: 0.023), but not with the other herpes virus infections (*P* value for IVW: 0.574 and 0.795; **Figure 3**). It appeared that subtype CD was also weakly associated with mononucleosis from the results, but this still requires further analysis (*P* value for IVW: 0.058; *P* value for Weighted median: 0.036; **Figure 4**). Here, it was evident that CD or UC had no causal relationship with herpes zoster (*P* value for IVW: 0.168 and 0.574, respectively). However, we found a suggestive association between subtype CD and chickenpox (*P* value for IVW: 0.038).

**Figure 5:**
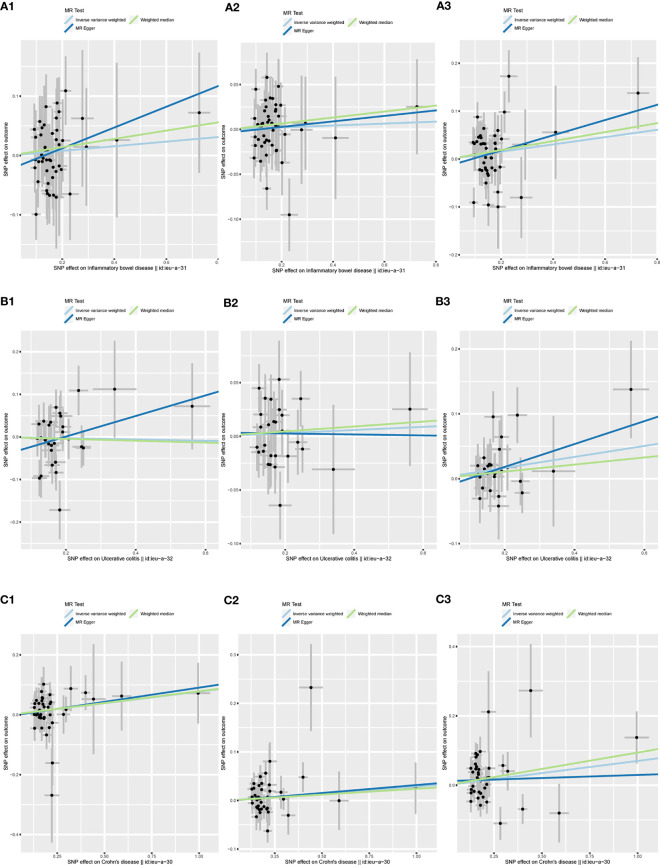
Scatter plots of the MR analysis. The slope of each line represents the effect estimated by an MR method. **(A1)** IBD on chickenpox; **(A2)** IBD on herpes zoster; **(A3)** IBD on mononucleosis; **(B1)** UC on chickenpox; **(B2)** UC on herpes zoster; **(B3)** UC on mononucleosis; **(C1)** CD on chickenpox; **(C2)** CD on herpes zoster; **(C3)** CD on mononucleosis. MR, mendelian randomization; IBD, inflammatory bowel disease; UC, ulcerative colitis; CD, Crohn’s disease.

**Figure 5** shows a scatter plot of the causal association between IBD and its two subtypes on herpes virus infections. **Table 1** lists the details of the sensitivity analysis. The forest plots of individual SNP causal effects are shown in **Supplementary Figure 1**; the leave-one-out plots are shown in **Supplementary Figure 2** and the funnel plots are shown in **Supplementary Figure 3**.

**Table 1:** Sensitivity analysis of MR.

Exposure	Outcome	MR Egger pleiotropy test		Heterogeneity test		MR-PRESSO outlier test	MR Steiger directionality test		
		Estimate	*P* value	Q	*P* value	outlier	Estimated variance explained in exposure	Estimated variance explained in outcome	Correct causal direction
IBD	chickenpox	-0.023	0.267	39.137	0.786	None	1.983	0.117	TRUE
	herpes zoster	-0.003	0.762	48.415	0.416	None	1.983	0.042	TRUE
	mononucleosis	-0.014	0.457	70.443	0.015	None	1.983	0.163	TRUE
UC	chickenpox	-0.047	0.182	36.342	0.134	None	1.354	0.117	TRUE
	herpes zoster	0.004	0.829	27.529	0.490	None	1.354	0.023	TRUE
	mononucleosis	-0.017	0.463	20.897	0.830	None	1.354	0.058	TRUE
CD	chickenpox	-0.004	0.839	30.002	0.875	None	3.555	0.191	TRUE
	herpes zoster	-0.001	0.934	45.84	0.243	None	3.555	0.090	TRUE
	mononucleosis	0.012	0.535	72.017	0.001	None	3.555	0.243	TRUE
chickenpox	IBD	-0.037	0.643	6.704	0.152	None	0.588	0.008	TRUE
	UC	-0.06	0.494	5.314	0.257	None	0.588	0.011	TRUE
	CD	-0.004	0.970	5.524	0.238	None	0.588	0.006	TRUE
herpes zoster	IBD	0.029	0.224	4.958	0.549	None	0.592	0.021	TRUE
	UC	0.046	0.132	7.614	0.268	None	0.592	0.070	TRUE
	CD	0.023	0.465	4.195	0.650	None	0.592	0.064	TRUE
mononucleosis	IBD	-0.026	0.275	6.333	0.706	None	1.265	0.007	TRUE
	UC	-0.057	0.085	6.133	0.727	None	1.265	0.018	TRUE
	CD	0.008	0.794	4.254	0.894	None	1.265	0.010	TRUE

### 3.2 Causal effects of herpes virus infections on IBD, UC, or CD

Herpes virus infections were used as exposure factors to analyze reverse causality on IBD, UC or CD. The *F* statistics for all SNPs were greater than 10, indicating that the bias of weak instrumental variables on the results of this study could be ignored. We also have no evidence of a causal relationship between genetically predicted herpes virus infections and the use of immunosuppressants (*P* value for IVW: 0.725 to 0.889, **Supplementary Table 8**). Therefore, the bias caused by immunosuppressants could also be negligible in the reverse MR analysis.

Based on the IVW approach, herpes virus infections (chickenpox, herpes zoster, and mononucleosis) did not show causal effects on IBD (*P* value: 0.503 to 0.888). In addition, we found no evidence linking herpes virus infections with UC (*P* value for IVW: 0.304 to 0.757). Herpes virus infections and CD were also not relevant (*P* value for IVW: 0.239 to 0.830). The results were consistent with estimates made using MR-Egger or Weighted median. Based on the results of the MR-Egger intercept test (*P* value: 0.085 to 0.970) and Cochran’s Q test (*P* value: 0.152 to 0.894), we found no evidence for the presence of pleiotropy and heterogeneity. The MR Steiger directionality test suggested that all causal directions were true, and the MR-PRESSO test did not identify outliers.

The forest plots and scatter plots of the genetically predicted herpes virus infections on IBD and its two subtypes are shown in **Supplementary Figures 4–7**. Details of the sensitivity analysis are presented **Table 1**. The forest plots of individual SNP causal effects are presented in **Supplementary Figure 8**; the leave-one-out plots are presented in **Supplementary Figure 9**, and the funnel plots are presented in **Supplementary Figure 10**.

## 4 Discussion

Our results demonstrated that genetically predicted total IBD was not associated with an increased risk of chickenpox (IVW: OR = 1.040; 95% CI = 0.952 to 1.136; *P* value = 0.390) or herpes zoster (IVW: OR = 1.011; 95% CI = 0.964 to 1.060; *P* value = 0.390). Genetically predicted total IBD had a borderline association with mononucleosis (IVW: OR = 1.079; 95% CI = 0.996 to 1.169; *P* value = 0.063). For subtypes of IBD, an association between UC and mononucleosis was suggested (IVW: OR = 1.088; 95% CI = 1.012 to 1.170; *P* value = 0.023), while for CD an association with chickenpox was suggested (IVW: OR = 1.081; 95% CI = 1.004 to 1.162; *P* value = 0.038). In addition, genetically predicted CD had a borderline association with mononucleosis (IVW: OR = 1.073; 95% CI = 0.998 to 1.153; *P* value = 0.058). Our results did not provide evidence for a causal association between UC (IVW: OR = 1.015; 95% CI = 0.964 to 1.068; *P* value = 0.574) or CD (IVW: OR = 1.029; 95% CI = 0.988 to 1.072; *P* value = 0.168) on herpes zoster. Herpes virus infections (chickenpox, herpes zoster, and mononucleosis) were not genetically associated with either IBD, UC, or CD.

Many medical literatures examined the association between IBD and herpes virus infections. A meta-analysis of six cohort studies involving 216,552 patients with IBD concluded that patients with IBD had an increased risk of herpes zoster (OR = 1.68; 95% CI = 1.53 to 1.84) (12). The study proceeded to analyze the effect of two subtypes of IBD on herpes zoster, and also found that UC (OR = 1.49; 95% CI = 1.34 to 1.65) and CD (OR = 1.67; 95% CI = 1.40 to1.98) were associated with an increased risk of herpes zoster. Another meta-analysis of seven cohort studies, which included over 1,000,000 participants, showed an association between UC (OR = 1.40; 95% CI = 1.31 to 1.50) and CD (OR = 1.74; 95% CI = 1.57 to 1.92) with herpes zoster (33). A retrospective cohort study involving 108,604 patients with IBD and 43,416 controls without IBD showed that the IBD cohort had a significantly increased risk of herpes zoster compared with the non-IBD cohort (34). In addition, Adams et al. reported an association between two subtypes of IBD and herpes zoster and chickenpox in a cross-sectional study using the Utilization Project Kids Inpatient Database (16). Compared to children with UC (herpes zoster: OR = 3.90; 95% CI = 1.98 to 7.67; chickenpox: OR = 4.25; 95% CI = 1.98 to 9.12), a stronger association was found in children with CD (herpes zoster: OR = 7.91; 95% CI = 5.60 to 11.18; chickenpox: OR = 12.75; 95% CI = 8.30 to 19.59). A population-based case-control study by Gearry et al. involving 653 patients with UC, 638 with CD and 600 controls found an association between CD and mononucleosis (OR = 1.64; 95% CI = 1.11 to 2.43), but no evidence of an association between UC and mononucleosis (35). The results of these reports show some discrepancies with our causal associations, which may be due to different methods of analysis. Observational or retrospective studies may be influenced by some unavoidable confounders that interfere with the estimation of exposure-outcome correlations. Observational or retrospective studies may be subject to unavoidable confounders that interfere with the estimation of exposure-outcome correlations and weaken the power of the findings to make precise causal decisions. This means that a direct causal relationship could not be proven, although one observational or retrospective study reported a potentially strong association. The MR analysis approach largely avoids the interference of potential confounders by introducing genetic variants, thereby providing a relatively precise estimate of causal associations.

Our study provides some evidence that the increased risk of chickenpox is associated with CD but not with UC; the opposite is true for mononucleosis, which is associated with UC but not with CD. There may be similarities in genetic background between different subtypes of the same disease, but there are still significant differences in immunity, individual genes, and genetics between the two subtypes of IBD (UC and CD) (36–38). More in-depth studies are needed in the future to analyze whether those differences originate from immune responses, environmental factors, genetic factors or changes in the intestinal flora.

The present study has three strengths. Firstly, to our knowledge, this is the first report to use large-scale GWAS data for a two-sample MR approach to assess the bidirectional causal association between IBD and herpes virus infections. Compared to previous observational and retrospective studies, this approach is less susceptible to the influence of confounders. Secondly, rigorous identification of IBD subtypes to avoid bias caused by the co-existence of two subtypes. Thirdly, a number of sensitivity analyses were carried out to assess the robustness of the results. Our study also has limitations. Selection of instrumental variables for herpes virus infections, we used looser thresholds. Although the *F* statistic indicated that the bias due to weak instrumental variables could be ignored, caution should be taken when interpreting this negative result. We matched selected SNPs to data in the phenoscanner database to avoid the effect of potential confounders. However, this method does not completely rule out the existence of pleiotropy, as the biological function of many genetic instrumental variables is unknown.

When interpreting our MR results, the following additional points should be considered. Firstly, it should be remembered that univariable MR analysis only reveals the overall effect of exposure-outcome, not direct effects. The pathway from exposure to outcome is extremely complex, especially in this study, as the use of medications may be a mediator of increased herpes virus infections in IBD. Although we analyzed the bidirectional cause-and-effect association between immunosuppressants and herpes virus infections to adjust for potential bias, we did not assess the effect of each individual immunosuppressant separately. Therefore, there is a possibility that the use of a particular immunosuppressant increases the risk of herpes virus infections, rather than the IBD disease itself, or the immunosuppressants as a whole. Secondly, it would be inappropriate to guide clinical interventions based on the results of the MR causal association, but it would help to screen for specific populations susceptible to chickenpox and mononucleosis. Thirdly, established cause-effect associations provide strong support for randomized controlled trials in real-world disease research, subject to ethical principles.

## 5 Conclusion

In conclusion, our study found no evidence of a causal association of IBD with chickenpox, herpes zoster, or mononucleosis. For subtypes of IBD, the increased risk of chickenpox is associated with CD but not with UC; the opposite is true for mononucleosis, which is associated with UC but not with CD. Neither subtype was associated with herpes zoster. More in-depth research is needed to elucidate the mechanisms of these causal associations.

## Data availability statement

The original contributions presented in the study are included in the article/**Supplementary Material**. Further inquiries can be directed to the corresponding authors.

## Author contributions

MZ designed the study and wrote the manuscript. WZ and LS analyzed and visualized the data. YX and YZ helped revise the manuscript. YX and YZ provided the funding. YZ supervised the study. All authors contributed to the article and approved the submitted version.
