# Supplementary material for: Causal association between inflammatory bowel disease and herpes virus infections: a two-sample bidirectional Mendelian randomization study
Source: Front Immunol. 2023 Jul 3;14:1203707. doi: 10.3389/fimmu.2023.1203707 (PMC10351388; doi:10.3389/fimmu.2023.1203707)
Supplement: Supplementary file 1 [file DataSheet_1.docx]

**Supplementary Figure 1.** Forest plot of the causal association of each SNP associated with IBD and its two subtypes on herpes virus infections.

**Supplementary Figure 2.** Leave-one-out analyses for the MR analysis of IBD and its two subtypes on herpes virus infections.

**Supplementary Figure 3.** Funnel plot for the MR analysis of IBD and its two subtypes on herpes virus infections.

**Supplementary Figure 4.** Estimates of the causal relationship between chickenpox and IBD and its two subtypes expressed as an odds ratio and 95% confidence interval.

**Supplementary Figure 5.** Estimates of the causal relationship between herpes zoster and IBD and its two subtypes expressed as an odds ratio and 95% confidence interval.

**Supplementary Figure 6.** Estimates of the causal relationship between secondary mononucleosis and IBD and its two subtypes expressed as an odds ratio and 95% confidence interval.

**Supplementary Figure 7.** Scatter plots for the MR analysis of herpes virus infections on IBD and its two subtypes.

**Supplementary Figure 8.** Forest plot of the causal association of each SNP associated with herpes virus infections on IBD and its two subtypes.

**Supplementary Figure 9.** Leave-one-out analyses for the MR analysis of herpes virus infections on IBD and its two subtypes.

**Supplementary Figure 10.** Funnel plot for the MR analysis of herpes virus infections on IBD and its two subtypes.


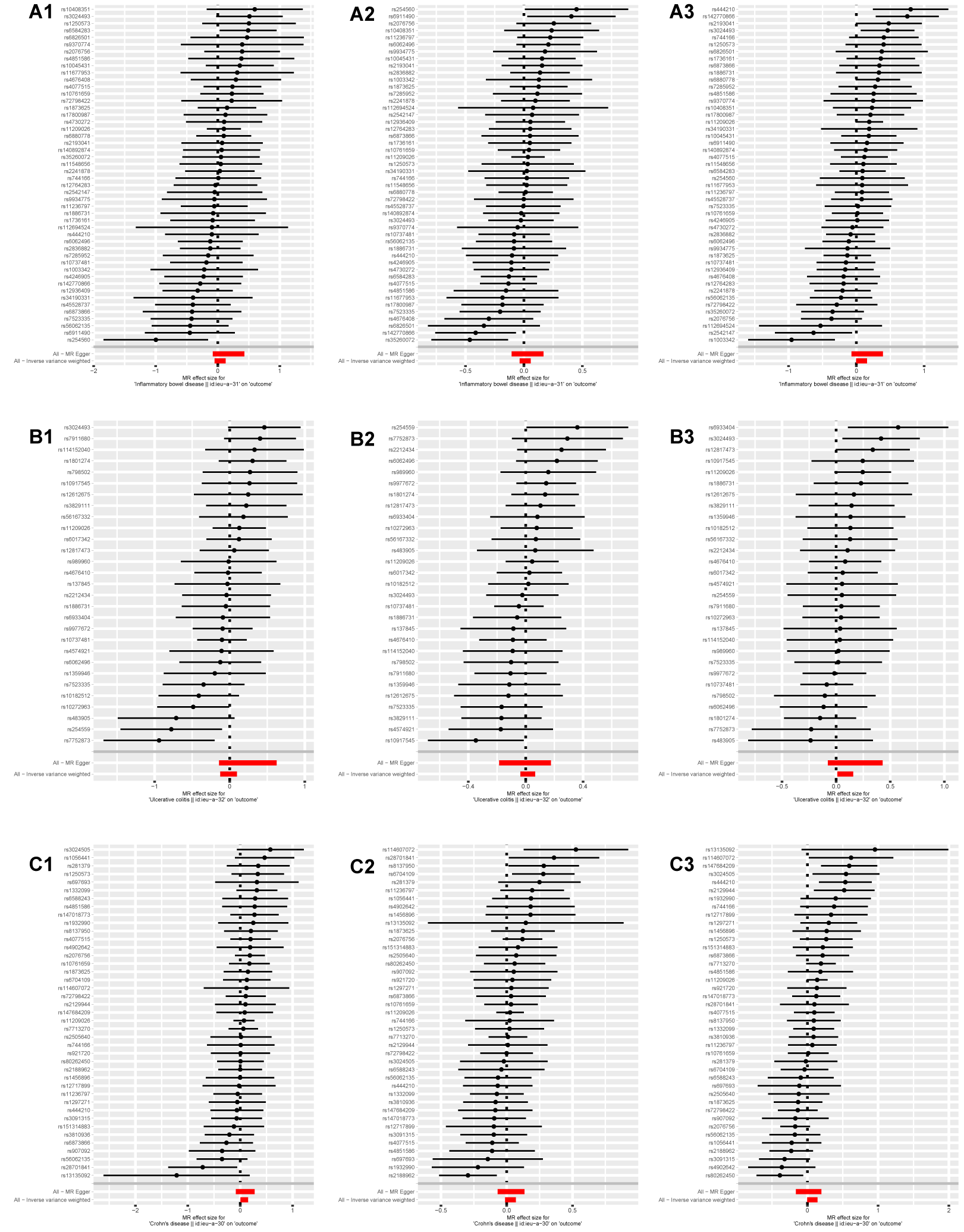


**Supplementary Figure 1.** Forest plot of the causal association of each SNP associated with IBD and its two subtypes on herpes virus infections. **(A1)** IBD on chickenpox; **(A2)** IBD on herpes zoster; **(A3)** IBD on mononucleosis; **(B1)** UC on chickenpox; **(B2)** UC on herpes zoster; **(B3)** UC on mononucleosis; **(C1)** CD on chickenpox; **(C2)** CD on herpes zoster; **(C3)** CD on mononucleosis. SNP, single-nucleotide polymorphism; IBD, inflammatory bowel disease; UC, ulcerative colitis; CD, Crohn's disease.


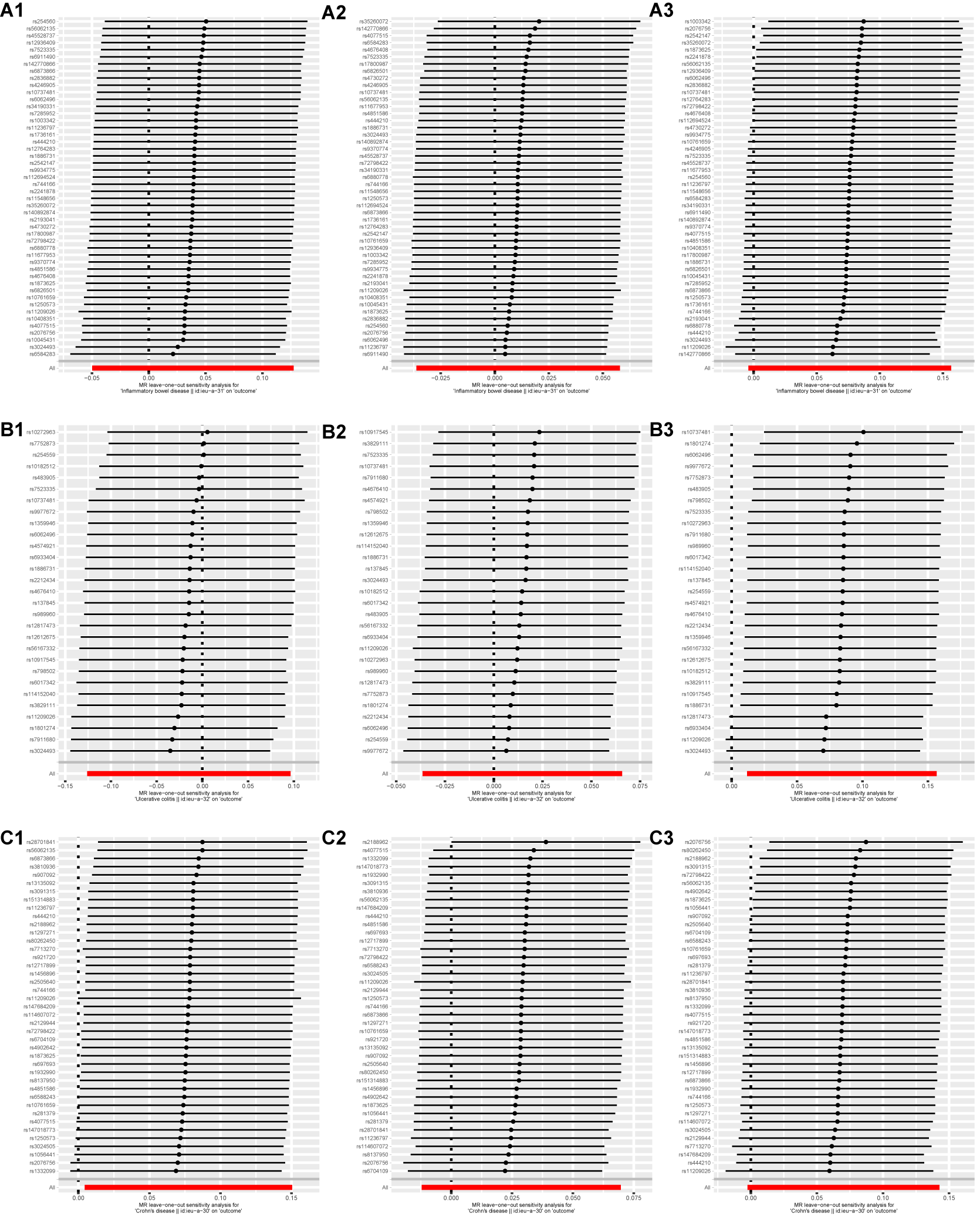


**Supplementary Figure 2.** Leave-one-out analyses for the MR analysis of IBD and its two subtypes on herpes virus infections. **(A1)** IBD on chickenpox; **(A2)** IBD on herpes zoster; **(A3)** IBD on mononucleosis; **(B1)** UC on chickenpox; **(B2)** UC on herpes zoster; **(B3)** UC on mononucleosis; **(C1)** CD on chickenpox; **(C2)** CD on herpes zoster; **(C3)** CD on mononucleosis. SNP, single-nucleotide polymorphism; IBD, inflammatory bowel disease; UC, ulcerative colitis; CD, Crohn's disease.


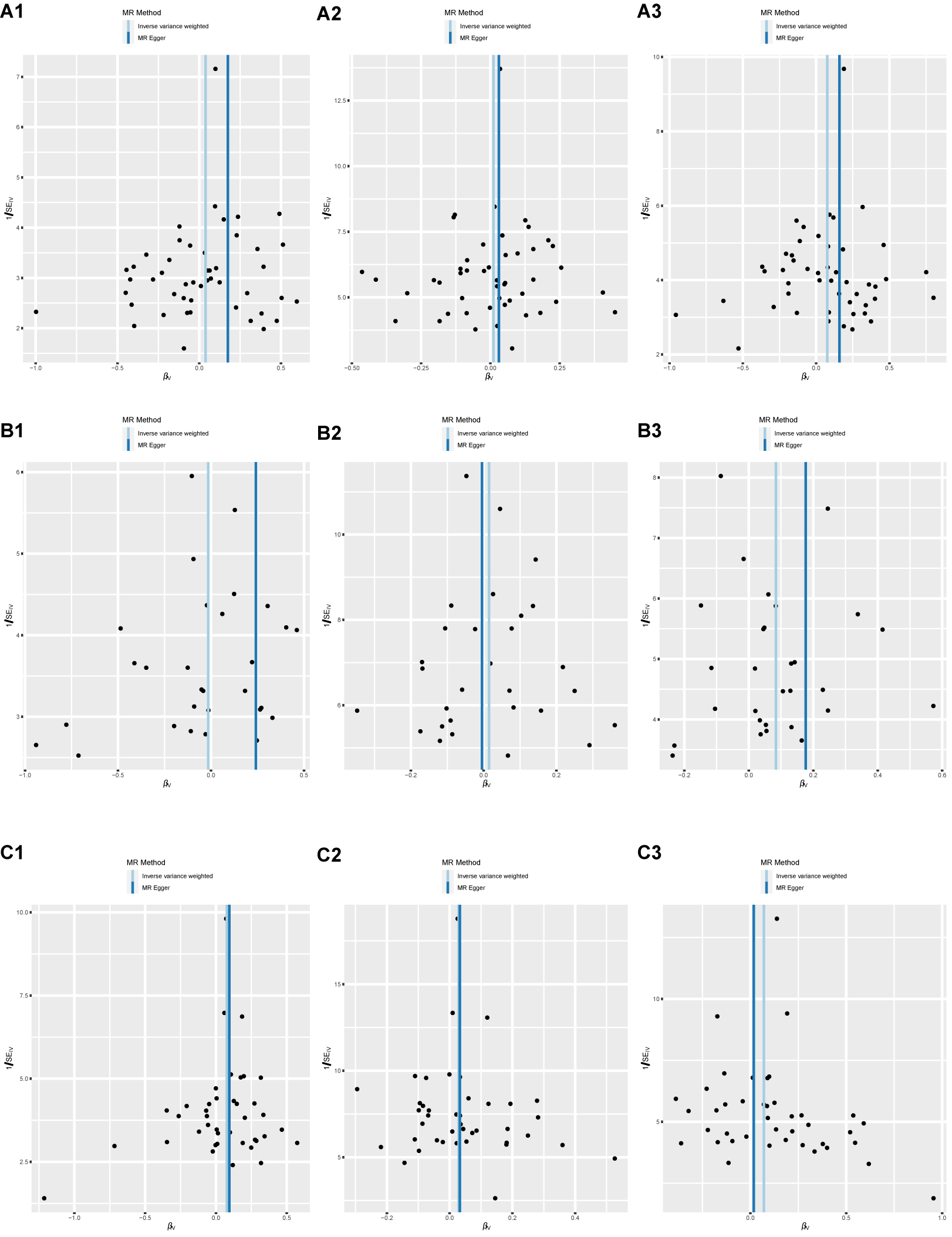


**Supplementary Figure 3.** Funnel plot for the MR analysis of IBD and its two subtypes on herpes virus infections. **(A1)** IBD on chickenpox; **(A2)** IBD on herpes zoster; **(A3)** IBD on mononucleosis; **(B1)** UC on chickenpox; **(B2)** UC on herpes zoster; **(B3)** UC on mononucleosis; **(C1)** CD on chickenpox; **(C2)** CD on herpes zoster; **(C3)** CD on mononucleosis. SNP, single-nucleotide polymorphism; IBD, inflammatory bowel disease; UC, ulcerative colitis; CD, Crohn's disease.


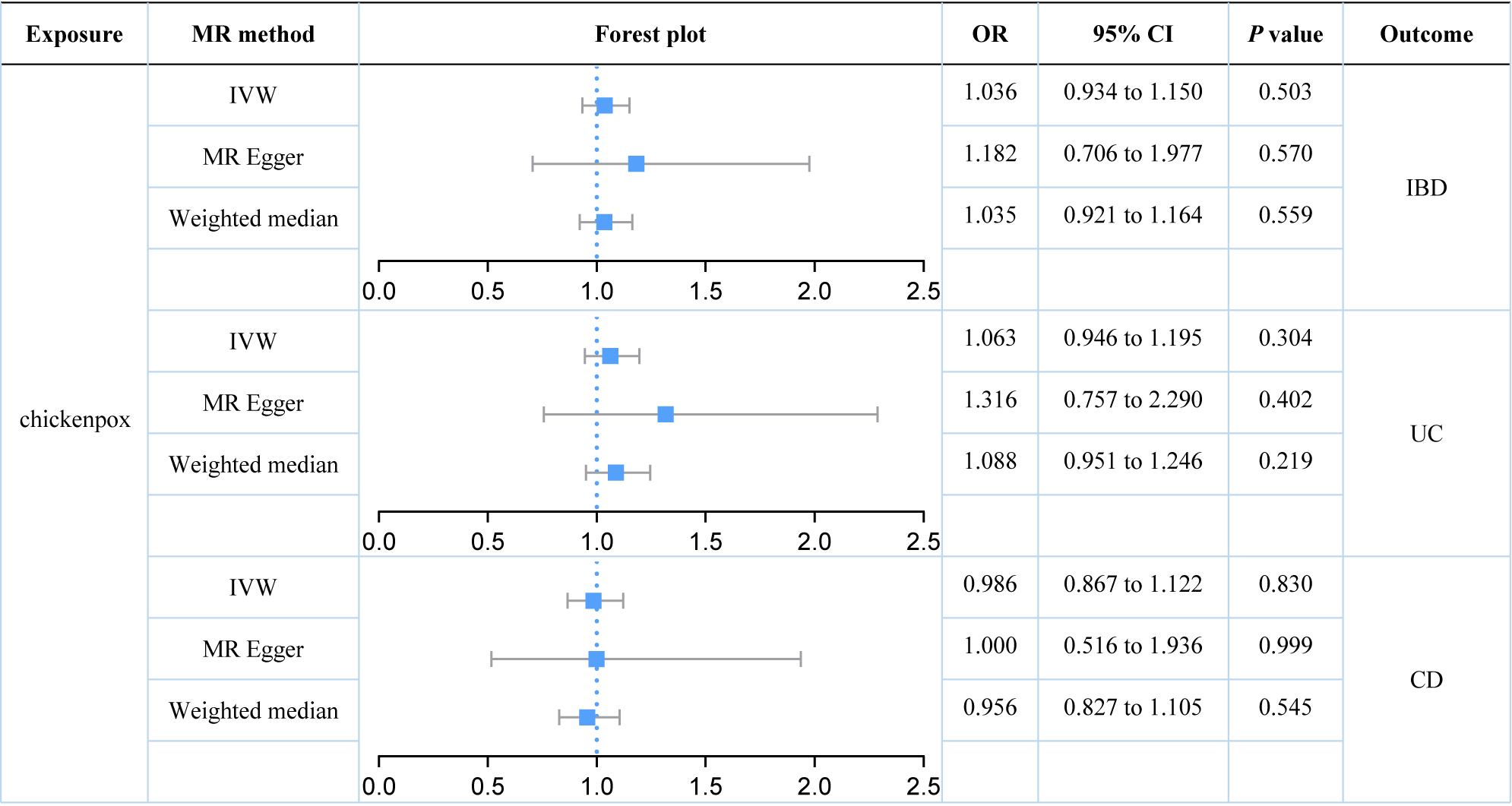


**Supplementary Figure 4.** Estimates of the causal relationship between chickenpox and IBD and its two subtypes expressed as an odds ratio (OR) and 95% confidence interval (CI). MR, mendelian randomization; IVW, inverse variance weighting; IBD, inflammatory bowel disease; UC, ulcerative colitis; CD, Crohn's disease.


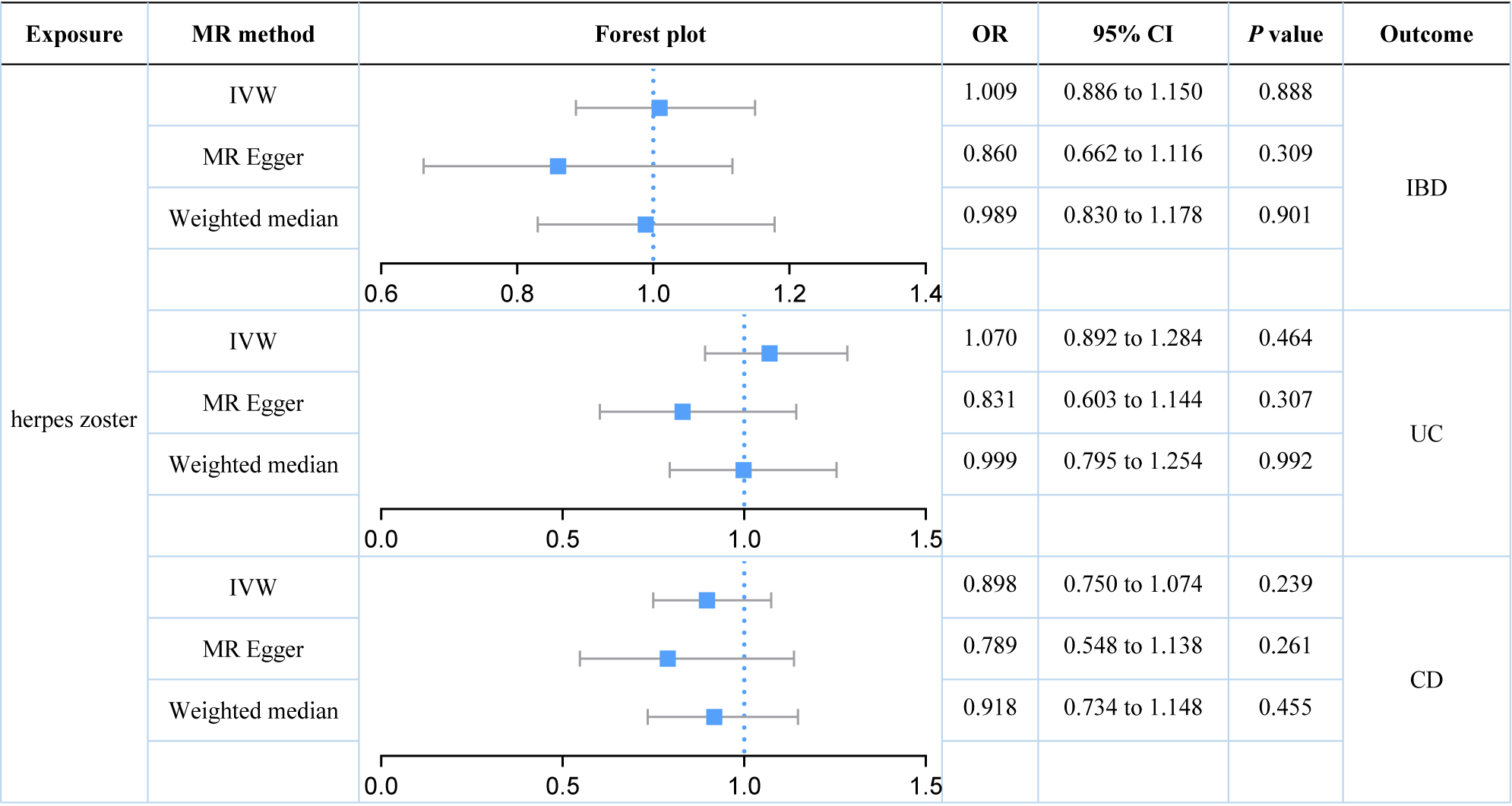


**Supplementary Figure 5.** Estimates of the causal relationship between herpes zoster and IBD and its two subtypes expressed as an odds ratio (OR) and 95% confidence interval (CI). MR, mendelian randomization; IVW, inverse variance weighting; IBD, inflammatory bowel disease; UC, ulcerative colitis; CD, Crohn's disease.


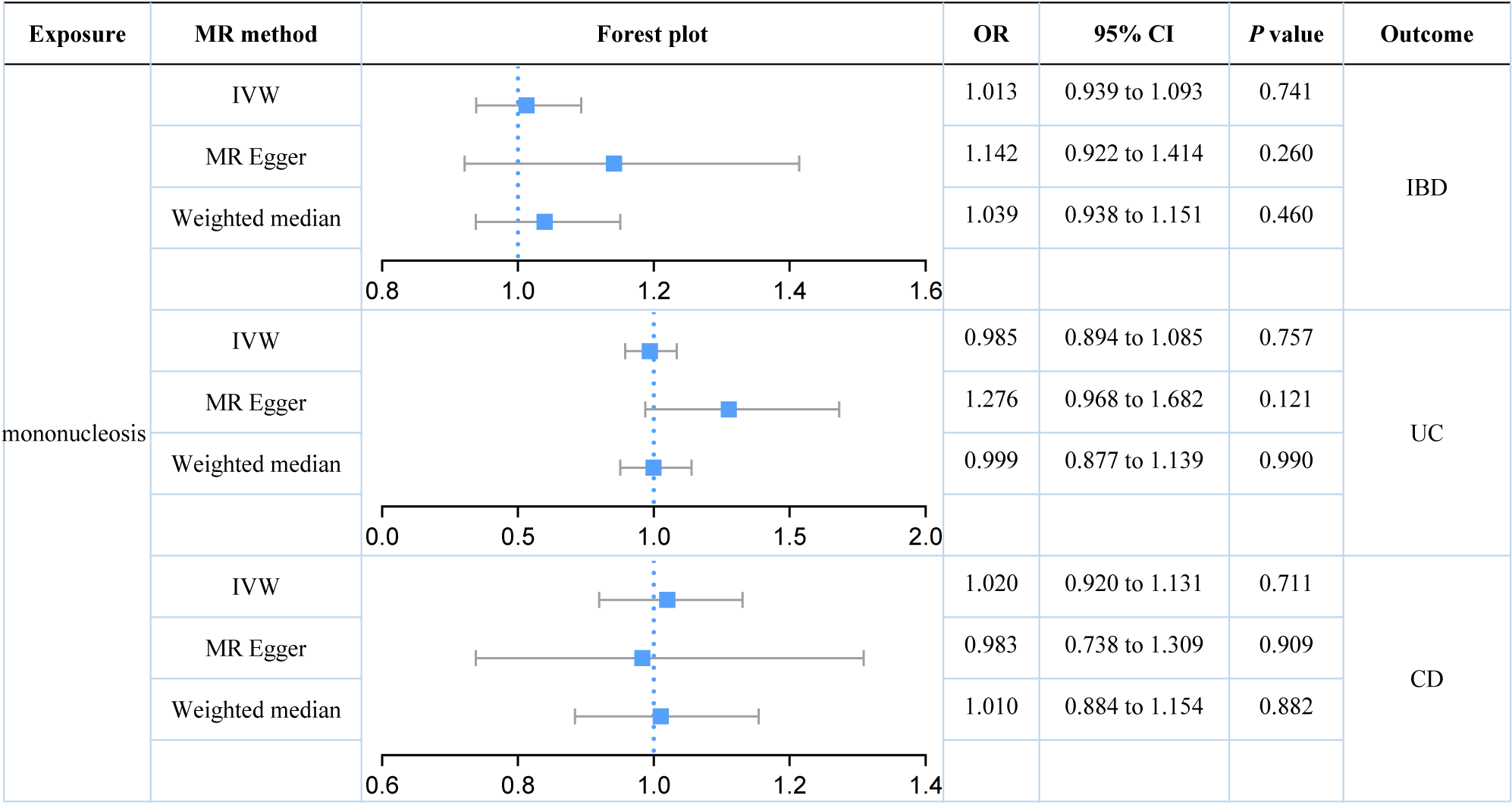


**Supplementary Figure 6.** Estimates of the causal relationship between mononucleosis and IBD and its two subtypes expressed as an odds ratio (OR) and 95% confidence interval (CI). MR, mendelian randomization; IVW, inverse variance weighting; IBD, inflammatory bowel disease; UC, ulcerative colitis; CD, Crohn's disease.


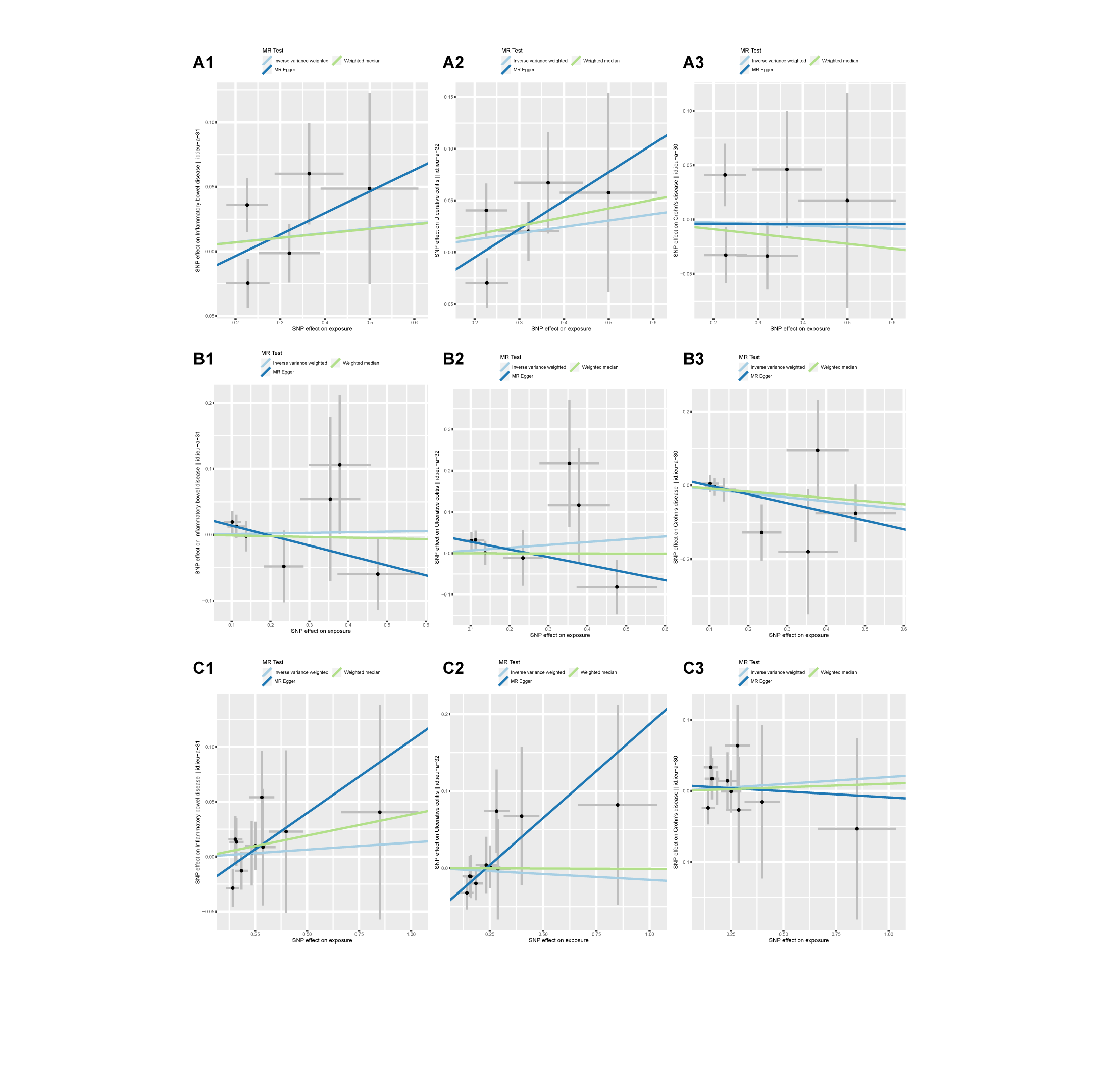


**Supplementary Figure 7.** Scatter plots for the MR analysis of herpes virus infections on IBD and its two subtypes. The slope of each line represents the effect estimated by an MR method. **(****A1)** chickenpox on IBD; **(A2)** chickenpox on UC; **(A3)** chickenpox on CD; **(B1)** herpes zoster on IBD; **(B2)** herpes zoster on UC; **(B3)** herpes zoster on CD; **(C1)** mononucleosis on IBD; **(C2)** mononucleosis on UC; **(C3)** mononucleosis on CD. MR, mendelian randomization; IBD, inflammatory bowel disease; UC, ulcerative colitis; CD, Crohn's disease.


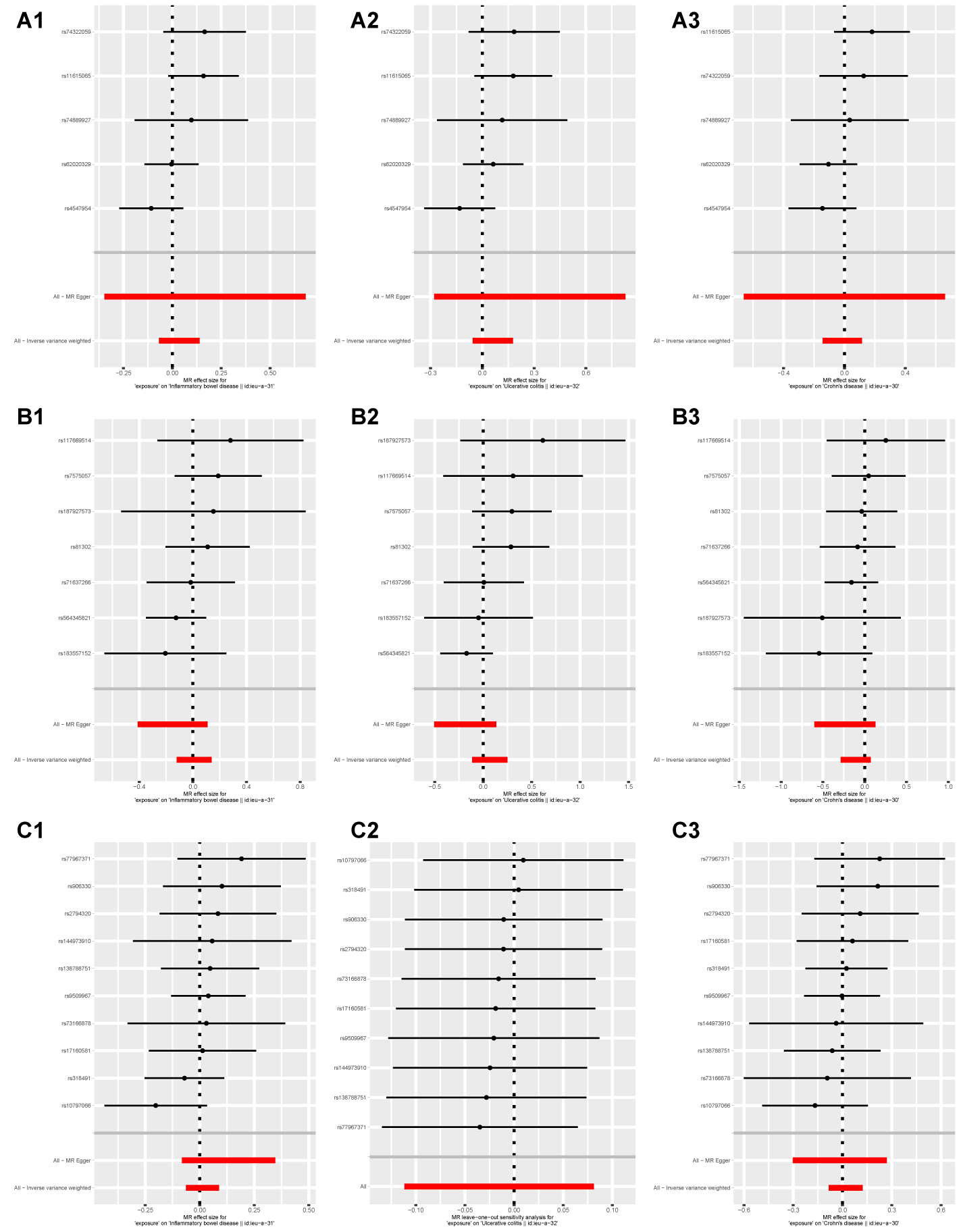


**Supplementary Figure 8.** Forest plot of the causal association of each SNP associated with herpes virus infections on IBD and its two subtypes. **(A1)** chickenpox on IBD; **(A2)** chickenpox on UC; **(A3)** chickenpox on CD; **(B1)** herpes zoster on IBD; **(B2)** herpes zoster on UC; **(B3)** herpes zoster on CD; **(C1)** mononucleosis on IBD; **(C2)** mononucleosis on UC; **(C3)** mononucleosis on CD. MR, mendelian randomization; IBD, inflammatory bowel disease; UC, ulcerative colitis; CD, Crohn's disease.


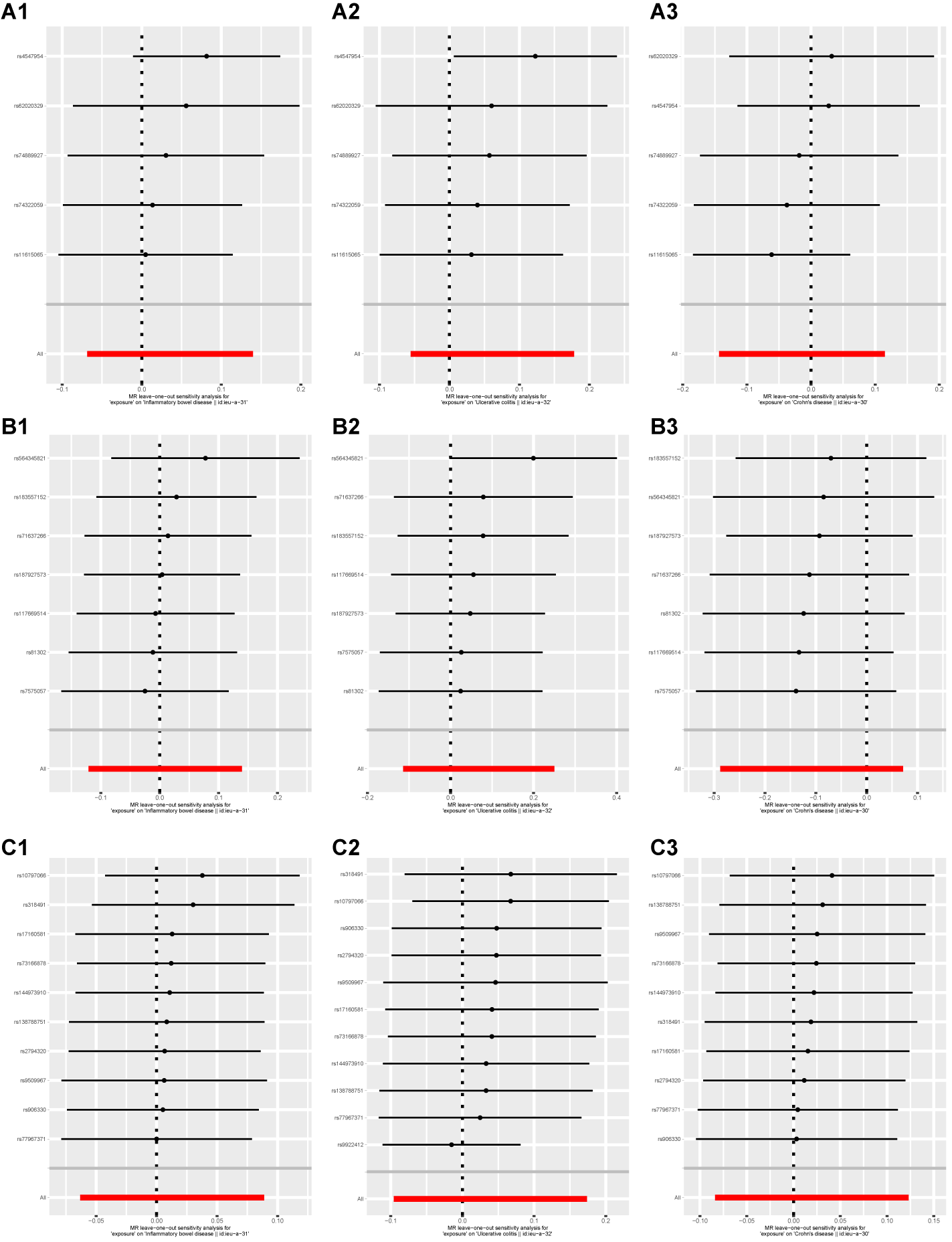


**Supplementary Figure 9.** Leave-one-out analyses for the MR analysis of herpes virus infections on IBD and its two subtypes. **(A1)** chickenpox on IBD; **(A2)** chickenpox on UC; **(A3)** chickenpox on CD; **(B1)** herpes zoster on IBD; **(B2)** herpes zoster on UC; **(B3)** herpes zoster on CD; **(C1)** mononucleosis on IBD; **(C2)** mononucleosis on UC; **(C3)** mononucleosis on CD. MR, mendelian randomization; IBD, inflammatory bowel disease; UC, ulcerative colitis; CD, Crohn's disease.


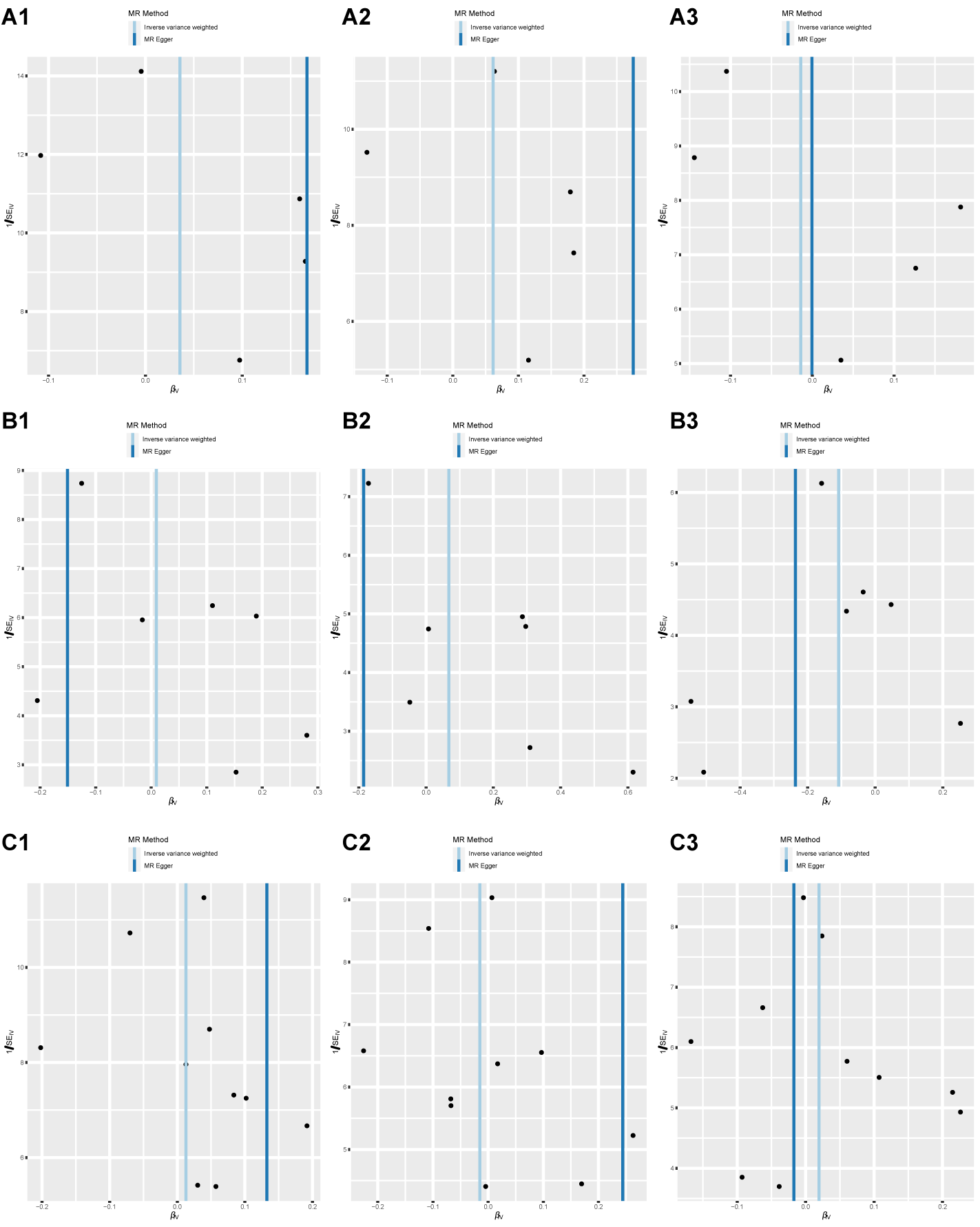


**Supplementary Figure 10.** Funnel plot for the MR analysis of herpes virus infections on IBD and its two subtypes. **(A1)** chickenpox on IBD; **(A2)** chickenpox on UC; **(A3)** chickenpox on CD; **(B1)** herpes zoster on IBD; **(B2)** herpes zoster on UC; **(B3)** herpes zoster on CD; **(C1)** mononucleosis on IBD; **(C2)** mononucleosis on UC; **(C3)** mononucleosis on CD. MR, mendelian randomization; IBD, inflammatory bowel disease; UC, ulcerative colitis; CD, Crohn's disease.
